# On the Asymptotic Capacity of Information-Theoretic Privacy-Preserving Epidemiological Data Collection

**DOI:** 10.3390/e25040625

**Published:** 2023-04-06

**Authors:** Jiale Cheng, Nan Liu, Wei Kang

**Affiliations:** 1 National Mobile Communications Research Laboratory, Southeast University, Nanjing 211189, China; 2 School of Information Science and Engineering, Southeast University, Nanjing 211189, China

**Keywords:** secure multiparty computation, data privacy, epidemiological data collection, asymptotic capacity

## Abstract

The paradigm-shifting developments of cryptography and information theory have focused on the privacy of data-sharing systems, such as epidemiological studies, where agencies are collecting far more personal data than they need, causing intrusions on patients’ privacy. To study the capability of the data collection while protecting privacy from an information theory perspective, we formulate a new distributed multiparty computation problem called privacy-preserving epidemiological data collection. In our setting, a data collector requires a linear combination of *K* users’ data through a storage system consisting of *N* servers. Privacy needs to be protected when the users, servers, and data collector do not trust each other. For the users, any data are required to be protected from up to *E* colluding servers; for the servers, any more information than the desired linear combination cannot be leaked to the data collector; and for the data collector, any single server can not know anything about the coefficients of the linear combination. Our goal is to find the optimal collection rate, which is defined as the ratio of the size of the user’s message to the total size of downloads from *N* servers to the data collector. For achievability, we propose an asymptotic capacity-achieving scheme when E<N−1, by applying the cross-subspace alignment method to our construction; for the converse, we proved an upper bound of the asymptotic rate for all achievable schemes when E<N−1. Additionally, we show that a positive asymptotic capacity is not possible when E≥N−1. The results of the achievability and converse meet when the number of users goes to infinity, yielding the asymptotic capacity. Our work broadens current researches on data privacy in information theory and gives the best achievable asymptotic performance that any epidemiological data collector can obtain.

## 1. Introduction

During any prevention and control period in an epidemic, strengthening the protection of personal information is conducive not only to safeguarding personal interests, but also better controlling the development of the epidemic. Differently from the collection of other homogeneous data, such as the large-scale labeled sample obtained in machine learning, the characteristics of medical or epidemiological data collection are reflected in the following aspects: (1) In order to establish a surveillance system for a dynamical group of people to collect syndromic data, the sample size is always changing [1,2]; (2) Data sharing and early response are critical in containing the spread of highly infectious diseases, such as COVID-19. This requires the data stored in the database to be updated to track real-time changes in symptoms, severity, or other disease-related patterns [3,4]; (3) Some of the epidemiological data, such as the locations of infected individuals, the blood oxygen saturation levels of patients with respiratory diseases after medication, or the physical condition monitoring data after viral infection, are related to the user’s privacy, so a “privacy-first” approach which uses dynamic identifiers and stores their data in a cryptographically secure manner is needed [5]. Hence, for these factors of epidemiological data collection, the key to ensuring the efficiency of information sharing and privacy protection is to maximize the balance between data privacy and collection rate in epidemic analysis.

While the data collection from public health authorities and the open-source access to researchers can provide convenience to epidemiologists, these strategies may also significantly intrude upon citizens’ privacy. Some of these individuals were affected by unwanted privacy invasion, and the ubiquitous data surveillance devices certainly exacerbate those concerns. Therefore, the responsible use of shared data and algorithms, the compliance with data protection regulations, and the appropriate respect for privacy and confidentiality have become important topics in epidemiological data collection [6,7]. In 2014, the Global System for Mobile Communications (GSM) Association outlined some privacy standards for data-processing agencies regarding mobile phone data collection in response to the Ebola virus [8]. Some other methods that effectively guarantee user privacy include: encryption mechanisms [9], the privacy-aware energy-efficient framework (P-AEEF) protocol [10], the differential privacy-based classification algorithm [11], and the spatio-temporal trajectory method [12]. Moreover, unauthorized agencies, malicious hackers, and unidentified attacks, such as traffic analysis attacks, fake-node injection, and selective forwarding, may also eavesdrop on users’ data under the current ambiguous and non-uniform collecting algorithms [13]. To avoid the leakage of information from malicious collecting servers or untrusted access, the privacy of data needs to be ensured during data storage and processing.

In line with ensuring the security and privacy of user data, epidemiological data collection also requires the protection of data collectors. In accessing public databases, various epidemiological investigation agencies have different requirements for data privacy. For example, many epidemiological surveys are conducted by governments, universities, research institutes, pharmaceutical companies, and private institutions. Access to public data may reveal these agencies’ data preferences, resulting in information leakage. Therefore, the private-preserving epidemiological data collection includes the user’s privacy regarding the storage and data collector, along with the data collector’s privacy in data storage.

In epidemiological modeling, many recent studies have shown that various models have a good fitting effect on the nature of epidemics, such as the Bayesian model [14] and deep learning models, including multi-head attention, long short-term memory (LSTM), and the convolutional neural network (CNN) [15]. Additionally, when it comes to privacy and security concerns, some work conducted in computer science, cryptography, and information theory provides handy tools to model and solve such problems. The privacy leakage was modeled in differential privacy [16], *k*-anonymity [17], *t*-closeness [18], interval privacy [19], etc. With those concerns and analysis, several studies in cryptography and information theory have focused on the issue of sharing messages to untrusted agencies, such as distributed linear separable computation [20,21,22], secured matrix multiplication [23,24,25], secure aggregation [26], participatory sensing [27], and private information retrieval [28,29,30,31]. Specifically, a cryptographical epidemiological model with data security and privacy called RIPPLE was analyzed [32]. This model enables the execution of epidemiological models based on the most-recent real contact graph while keeping all the users’ information private. As an extension to the model, the data collector uses the sum private information retrieval (PIR) schemes to obtain the statistical data, which is described as the sum of a set of elements from a database.

Inspired by the cryptographical epidemiological model in [32], we propose an information-theoretic privacy-preserving epidemiological-data-collection problem, described as follows. We have a large and changing number of users sharing their real-time epidemiological data to *N* servers. The server will update its database once new data are uploaded. The storage of those users’ data is also open to all authorized data collectors. For any data collector who desires only some statistical feature, rather than all the data, it directly retrieves the statistic from *N* servers. We designed the protocol of the interaction between the *N* servers and the data collector so that when all *N* servers honestly answer the queries from the data collector, then the desired statistical data can be correctly decoded at the data collector. To simplify the problem, we assume the desired statistics to be the linear combinations of users’ personal data. The privacy of this model is reflected in the following three aspects: (1) The privacy of users’ data against the data collector: after downloading all answers from the servers, the data collector can decode the desired data without learning anything about the irrelevant details of the users’ personal information. (2) The privacy of users’ data against servers: for any user successfully sharing the data to all *N* servers, the personal information of the user is still confidential, even if of any up to E(E<N) servers collude. (3) The privacy of the data collector’s preference against servers: the protocol between data collector and servers should promise that any single server cannot know any preference of the desired statistical data from the query generated by the data collector. In our paper, we take the above privacy concerns into consideration and analyze the data collector’s ability of privately receiving the shared data, with respect to the number of symbols it needs to download.

The remainder of the paper is organized as follows: In Section 2, we introduce an information-theoretic description of the privacy-preserving epidemiological-data-collection problem, and we define the communication rate and capacity of our problem. In Section 3, we give a closed-form expression for the asymptotic capacity, which is the main result of the paper. In Section 4, we derive a converse proof for E<N−1, which provides an upper bound on the asymptotic capacity when the number of users tends to infinity. An asymptotically capacity-achieving scheme using the technique of cross-subspace alignment when E<N−1 is given in Section 5, and in Section 6, we prove that the problem is not asymptotically achievable when E=N−1. Finally, in Section 7, we summarize our results and suggest some open problems in this field.

## 2. System Model

**Figure 1:**
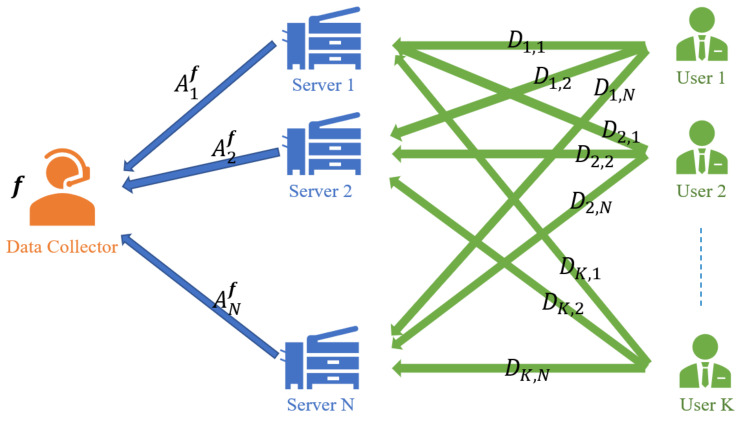
The secure, privacy-preserving epidemiological-data-collection problem.

We formulate the privacy-preserving epidemiological-data-collection problem over a typical distributed, secure computation system, in which there are *K* users, *N* servers, and a data collector; and the number of users ($K\in N_{+}$) is a large-enough integer. Here and throughout the paper, we assume that all of the random symbols in our system are generated by a large-enough finite field $F_{q}$, and we standardize the entropy of any uniformly distributed symbol to be 1 by taking the term log in the entropy, conditional entropy, and mutual information to be base *q*. The model is depicted in Figure 1.

Let $W_{1},\cdots ,W_{K}$ be *K* independent messages, where $W_{k},k\in [1:K]$ denotes the epidemiological data of user *k* and $W_{k}\in GF_{q}^{L\times 1},L\in N_{+}$ is an L×1 vector with *L* i.i.d. uniform symbols from the finite field $GF_{q}$—i.e.,

(1) $$\begin{array}{cc} \; & H\left(W_{\left[1:K\right]}\right)=\sum_{k=1}^{K}H\left(W_{k}\right),\\ \; & H\left(W_{k}\right)=L,\;\forall k\in \left[1:K\right]. \end{array}$$

The data-collection problem contains two phases: the upload phase and the computation phase. The upload phase starts when the user is required to update its data to each of the *N* servers. Before uploading, user *k* knows nothing about the contents of other users’ epidemiological data. While uploading their personal data, the users would like to keep his/her message private against *E*(E≤N,E∈N) colluding servers; i.e., any of up to *E* servers will learn nothing about the messages uploaded by *K* users. For any n∈[1:N], let $D_{k,n}\in D$ denote the uploaded message from the *k*-th user to the *n*-th server. We have the following equality called the privacy constraint of the users against *E* servers:

(2) $$\begin{array}{c} I\left(D_{k,E};W_{k}\right)=0,\;\forall k\in \left[1:K\right],\forall E\subseteq \left[1:N\right],\left|E\right|\leq E. \end{array}$$

For any k∈[1:K], let $Z_{k}\in Z_{k}$ denote a random variable privately generated by user *k*, and its realization is not known to any of the *N* servers. User *k* utilizes the user-side randomness $Z_{k}$ to encipher the message $W_{k}$; i.e., for any user *k*, there exist *N* functions ${\left\{d_{k,n}\right\}}_{n\in [1:N]}$ such that $d_{k,n}\left(W_{k},Z_{k}\right)=D_{k,n}$, where $d_{k,n}:GF_{q}^{L}\times Z_{k}\to D$ is the encoding function from the user *k* to the server *n*. We have

(3) $$\begin{array}{c} H\left(D_{k,[1:N]}|W_{k},Z_{k}\right)=0,\;\forall k\in \left[1:K\right]. \end{array}$$

When the servers receive the updated data from users, the old contents of the server will be replaced with the new contents, and all servers will be ready for the computation phase and allow the access of data updated by data collectors.

The computation phase starts when a data collector would like to compute statistical data of the current epidemiological database. To simplify our problem, we only consider statistical data to be a linear combination of all messages $W_{\left[K\right]}$. Let $W^{f}$ and f be the statistical data and the corresponding coefficient vector. Then, we have

(4) $$\begin{array}{c} W^{f}=f^{T}\left[\begin{array}{c} W_{1}\\ \vdots \\ W_{K} \end{array}\right]=\sum_{k=1}^{K}f_{k}W_{k}, \end{array}$$

where $W^{f}\in GF_{q}^{L}$ has the same number of symbols with the message length, and $f\in GF_{q}^{K}$ contains *K* elements in the finite field $GF_{q}$. In our setting, the coefficient vector f only contains the preference of the data collector among *K* user’s epidemiological data records. Therefore, the value of f does not depend on the users’ messages $W_{\left[1:K\right]}$ or the storage of *N* servers: ${\left\{D_{k,n}\right\}}_{k\in [1:K],n\in [1:N]}$.

Let $Z^{\prime }\in Z$ denote a random variable privately generated by the data collector, and its realization is not available to any of the *N* servers and *K* users. In the computation phase, the data collector with its preference vector f utilizes the randomness $Z^{\prime }$ to generate its queries to *N* servers. Assume that the query from the data collector to the *n*-th server is denoted as $Q_{n}^{f}\in Q_{n}$. Then, the data collector uses the strategy *g* to map the randomness and the coefficient to the queries, such that

$$\begin{array}{c} g\left(f,Z^{\prime }\right)={\left\{\begin{array}{c} Q_{1}^{f}\;Q_{2}^{f}\;\cdots Q_{N}^{f} \end{array}\right\}}^{T}, \end{array}$$

where $g:GF_{q}^{K}\times Z\to \prod_{n=1}^{N}Q_{n}$ is the encoding function from the data collector to the servers. Hence, we have

(5) $$\begin{array}{c} H(Q_{\left[1:N\right]}^{f}|Z^{\prime },f)=0. \end{array}$$

Since the randomness $Z^{\prime }$ and queries $Q_{\left[1:N\right]}^{f}$ are generated privately, and the user-side data and randomness $W_{\left[1:K\right]},Z_{\left[1:K\right]}$ are already known before the computation phase starts, the data collector has no knowledge of $W_{\left[1:K\right]},Z_{\left[1:K\right]}$ when the queries $Q_{\left[1:N\right]}^{f}$ are generated. Thus, we have

(6) $$\begin{array}{c} I(W_{\left[1:K\right]},Z_{\left[1:K\right]};Q_{\left[1:N\right]}^{f},Z^{\prime })=0. \end{array}$$

Upon receiving the query $Q_{n}^{f}$, the *n*-th server will send an answer $A_{n}^{f}$ back to the data collector according to the storage of the *n*-th server. We assume that there is no drop-out in the model—i.e., each server *n* will successfully return its answer to the data collector. Let $A_{n}^{f}\in A_{n}$ be the answer from the *n*-th server to the data collector, and then for any n∈[1:N], there exists a deterministic function $a_{n}^{f}:Q_{n}\times D^{K}\to A_{n},$ such that $A_{n}^{f}=a_{n}^{f}\left(Q_{n}^{f},{\left[\begin{array}{c} D_{1,n}\;D_{2,n},\cdots ,D_{K,n} \end{array}\right]}^{T}\right),\;\forall n\in \left[1:N\right]$. Hence,

(7) $$\begin{array}{c} H\left(A_{n}^{f}|Q_{n}^{f},D_{[1:K],n}\right)=0,\;\forall n\in \left[1:N\right]. \end{array}$$

We would like to design a scheme $\phi ={\left\{d_{k,n},g,a_{n}^{f}\right\}}_{k\in \left[1:K\right],n\in \left[1:N\right],f\in GF_{q}^{K}}$ in the upload and computation phases so that the following three constraints can be achieved. Firstly, the data collector is able to reconstruct the desired message from all the information it has received, which we call the decodability constraint. Let ψ be the reconstruction function of the data collector, where $\psi :\prod_{n=1}^{N}A_{n}\times \prod_{n=1}^{N}Q_{n}\times GF_{q}^{K}\times Z\to GF_{q}^{L}$. We have

(8) $$\begin{array}{c} {\hat{W}}^{f}=\psi \left(A_{\left[1:N\right]}^{f},Q_{\left[1:N\right]}^{f},f,Z^{\prime }\right). \end{array}$$

Let $P_{e}$ be the probability of decoding error achieved by a given scheme ϕ and decoding function ψ. We obtain

$$\begin{array}{c} P_{e}\left(\phi ,\psi \right)=\max_{f}\mathrm{Pr}\left\{{\hat{W}}^{f}\neq W^{f}\right\}. \end{array}$$

According to Fano’s inequality, the decodability constraint is equivalent to

(9) $$\begin{array}{cc} H(W^{f}|A_{\left[1:N\right]}^{f},Q_{\left[1:N\right]}^{f},Z^{\prime })= & o\left(L\right),\;\forall f\in GF_{q}^{N}, \end{array}$$

when $P_{e}\to 0$, where o(L) denotes any possible function $h:N_{+}\to R$ of *L* satisfying $\lim_{L\to \infty }\frac{h(L)}{L}=0$.

The second constraint guarantees the data privacy of *K* users against the data collector. The privacy leakage to the data collector is unavoidable, as the data collector must learn some statistical data of the users. This constraint requires that the data collector can learn nothing about the information of the *K* users other than the desired data $W^{f}$. We have

(10) $$\begin{array}{cc} I(W_{\left[1:K\right]};A_{\left[1:N\right]}^{f},Q_{\left[1:N\right]}^{f},Z^{\prime }|W^{f})= & 0. \end{array}$$

To protect the privacy of data collector, the third constraint requires the coefficient vector f of the data collector to be indistinguishable from the perspective of each server; i.e., for any different (linearly independent) coefficient vectors f and $f^{\prime }$ from the same scheme ϕ, the queries to every single server are identically distributed, so that any server cannot deduce f merely from the query and storage without communicating to other servers. Hence, we have

(11) $$\begin{array}{c} \left(A_{n}^{f},Q_{n}^{f},W_{\left[1:K\right]}\right)∼\left(A_{n}^{f^{\prime }},Q_{n}^{f^{\prime }},W_{\left[1:K\right]}\right),\;\forall f,f^{\prime }\;\mathrm{linear}\;\mathrm{independent} \end{array}$$

where A∼B means that the random variables *A* and *B* have the same distribution. This constraint is called the privacy constraint of the data collector against non-colluding servers.

The reason why in the upload phase, we consider up to *E* servers may collude, and in the computation phase, we consider non-colluding servers to be defined by the following: the upload phase and the computation phase do not always occur at the same time. For example, the users are required to upload their epidemiological data on a regular basis, and the data collector may start his/her queries of certain statistics at relatively random times. Due to the dynamic topology of the servers, the numbers of colluding servers may be different during the uploading phase and the computation phase. Our work assumes that the servers are non-colluding in the computation phase, since the servers may be more interested in the epidemiological data than the data collector’s interest. If E=1, then we have a model where the servers are non-colluding in both the upload phase and the computation phase.

For any scheme $\phi ={\left\{d_{k,n},g,a_{n}^{f}\right\}}_{k\in \left[1:K\right],n\in \left[1:N\right],f\in GF_{q}^{K}}$ that satisfies the above decodability constraint, i.e., (9), and the privacy constraints, i.e., the privacy constraint of the users against *E* servers (2), the privacy constraint of the users against the data collector (10), and the privacy constraint of the data collector against the non-colluding servers (11), its communication rate is characterized by the number of symbols the data collector decodes per download symbol—i.e.,

(12) $$\begin{array}{c} R:=\frac{L}{\sum_{n=1}^{N}H\left(A_{n}^{f}\right)}. \end{array}$$

It is worth noticing that *R* is not a function of f due to (11).

A rate *R* is said to be (ϵ-error) achievable if there exists a sequence of schemes indexed by *L* with their communication rate less than or equal to *R* where the probability of error $P_{e}$ goes to zero as L→∞. The ϵ-error capacity of this random secure aggregation problem is defined as the supremum of all ϵ-error achievable rates, i.e., C:=supR, where the supremum is over all possible ϵ-error achievable schemes.

## 3. Main Result

For the information-theoretical framework of our privacy-preserving epidemiological-data-collection problem presented in Section 2, our main result is the characterization of the asymptotic capacity when K→∞ for any $N\in N_{+}$ and E∈N.

To begin with, we show that the problem is infeasible when N=1 or N=E, since in these cases, some constraints of our setting contradict each other, and no scheme can satisfy all the constraints. Firstly, when there is only one server, the privacy of users against the data collector and the privacy of data collector against servers will conflict with each other. The reason is as follows. First, according to (11), the answer *A* will be given that for two different $f,f^{\prime }$, the distribution of *A* is the same. Second, the decodability (9) guarantees that $W^{f}$ can be derived from *A*. Then, $W^{f^{\prime }}$ can be derived from *A*, which contradicts the privacy of users against the data collector.

Moreover, when E=N, the decodability constraint and the privacy of users against the servers will conflict with each other. The reason is as follows. First, the answers $A_{\left[1:N\right]}^{f}$ from *N* servers are given by the queries $Q_{\left[1:N\right]}^{f}$ and the storage $D_{\left[1:K],[1:N\right]}$ according to (7), so there is a Markov chain $W_{\left[1:K\right]}\to \left(Q_{\left[1:N\right]}^{f},D_{\left[1:K],[1:N\right]}\right)\to A_{\left[1:N\right]}^{f}$. Second, when E=N, $Q_{\left[1:N\right]}^{f}$ and $D_{[1:K],n}$ are independent from $W_{\left[1:K\right]}$ according to (6) and (2), which contradicts the decodability constraint as $A_{\left[1:N\right]}^{f}$ is independent from the database $W_{\left[1:K\right]}$. Therefore, no scheme can simultaneously satisfy all the constraints in a single-server or E=N scenario, and there does not exist a positive capacity.

The following theorem gives the asymptotic capacity of private-preserving epidemiological data collection for an infinitely large *K*, where we have N≥2,E<N servers.

**Theorem** **1.** *Consider E as a non-negative number, and there are N≥2 servers. When E<N, and the number of users K→∞, the asymptotic capacity of the secure privacy-preserving epidemiological-data-collection problem is*

(13) $$\begin{array}{c} \lim_{K\to \infty ,L\to \infty }C=\left\{\begin{array}{cc} \frac{N-E-1}{N},\; & \;\mathrm{if}\;E<N-1\\ 0,\; & \;\mathrm{otherwise}\; \end{array}\right.. \end{array}$$

When E<N−1, the converse proof of Theorem 1 will be given in Section 4, and the achievability proof will be given in Section 5 for any finite $K\in N_{+}$. When K→∞, our scheme in Section 5 remains achievable at the same rate, and the performance of the achievability and converse will meet. When E=N−1, the proof of the zero asymptotic capacity is given in Section 6.

**Remark** **1.** *From Theorem 1, we can see a threshold in the asymptotic capacity on the number of the maximized possible colluding servers E. When E≥N−1, there is no scheme with a positive asymptotic capacity; when E<N−1, the asymptotic capacity is a decreasing function of E. When N approaches infinity while E is a constant, the asymptotic capacity approaches one.*

**Remark** **2.** *When the number of users K is a finite integer, the achievability and converse results do not always meet. From our converse proof in Section 4, the upper bound we give for finite K depends on the value of K. However, the performance (i.e., achievable rate) of our scheme in Section 5 is irrelevant to K, even though the scheme we propose is different for the finite $K\in N_{+}$. How to close the gap between the upper and lower bounds when K is finite is still an open problem.*

## 4. Proof of Theorem 1: Converse When E<N−1

We give the converse proof of Theorem 1 when E<N−1 in this section. The converse allows any feasible scheme ϕ, and we give an upper bound over the rates of all possible schemes. We start with the following lemma, which states an iterative relationship among the number of linear combinations of the users’ messages.

**Lemma** **1.** *Consider K linear independent vectors $f_{1},f_{2},\cdots ,f_{K}\in GF_{q}^{K}$. Let E be a set and E⊆[1:N], |E|=E. We have*

(14) $$\begin{array}{cc} \; & H(A_{[1:N]/E}^{f_{k}}|W^{f_{1}},\cdots ,W^{f_{k}},D_{[1:K],E},Q_{\left[1:N\right]}^{f_{k}},Z^{\prime })\\ \; & \geq \frac{L}{N-E}+\frac{1}{N-E}H\left(A_{[1:N]/E}^{f_{k+1}}|W^{f_{1}},\cdots ,W^{f_{k+1}},D_{[1:K],E},Q_{\left[1:N\right]}^{f_{k+1}},Z^{\prime }\right)-o\left(L\right). \end{array}$$

**Proof.**  According to our problem setting, we have

$$\begin{array}{cc} \; & \left(N-E\right)H\left(A_{[1:N]/E}^{f_{k}}|W^{f_{1}},\cdots ,W^{f_{k}},D_{[1:K],E},Q_{\left[1:N\right]}^{f_{k}},Z^{\prime }\right) \end{array}$$

(15) $$\begin{array}{cc} \geq & \sum_{n\in [1:N]/E}H\left(A_{n}^{f_{k}}|W^{f_{1}},\cdots ,W^{f_{k}},D_{[1:K],E},Q_{\left[1:N\right]}^{f_{k}},Z^{\prime }\right) \end{array}$$

(16) $$\begin{array}{cc} = & \sum_{n\in [1:N]/E}H\left(A_{n}^{f_{k+1}}|W^{f_{1}},\cdots ,W^{f_{k}},D_{[1:K],E},Q_{\left[1:N\right]}^{f_{k+1}},Z^{\prime }\right)\\ \geq & H(A_{[1:N]/E}^{f^{\prime }}|W^{f},D_{[1:K],E},Q_{\left[1:N\right]}^{f^{\prime }},Z^{\prime }) \end{array}$$

(17) $$\begin{array}{cc} = & H\left(A_{[1:N]/E}^{f^{\prime }}|W^{f},D_{[1:K],E},Q_{\left[1:N\right]}^{f^{\prime }},Z^{\prime }\right)+H\left(W^{f^{\prime }}|A_{[1:N]/E}^{f^{\prime }},W^{f},D_{[1:K],E},Q_{\left[1:N\right]}^{f^{\prime }},Z^{\prime }\right)-o\left(L\right)\\ = & H\left(W^{f^{\prime }}|W^{f},D_{[1:K],E},Q_{\left[1:N\right]}^{f^{\prime }}\right)+H\left(A_{[1:N]/E}^{f^{\prime }}|W^{f^{\prime }},W^{f},D_{[1:K],E},Q_{\left[1:N\right]}^{f^{\prime }},Z^{\prime }\right)-o\left(L\right) \end{array}$$

(18) $$\begin{array}{cc} = & L+H\left(A_{[1:N]/E}^{f^{\prime }}|W^{f^{\prime }},W^{f},D_{[1:K],E},Q_{\left[1:N\right]}^{f^{\prime }},Z^{\prime }\right)-o\left(L\right), \end{array}$$

where (15) holds because of the non-negativity of the following conditional entropy; i.e.,

$$\begin{array}{c} H\left(A_{\left[1:N]/(E\cup \{k\}\right)}^{f_{k}}|A_{n}^{f_{k}},W^{f_{1}},\cdots ,W^{f_{k}},D_{[1:K],E},Q_{\left[1:N\right]}^{f_{k}},Z^{\prime }\right)\geq 0,\;\forall n\in \left[1:N\right]/E, \end{array}$$

and (16) holds because of (11). The equality in (17) follows due to the fact that

$$\begin{array}{c} H\left(W^{f^{\prime }}|A_{[1:N]/E}^{f^{\prime }},W^{f},D_{[1:K],E},Q_{\left[1:N\right]}^{f^{\prime }},Z^{\prime }\right)=H\left(W^{f^{\prime }}|A_{[1:N]/E}^{f^{\prime }},W^{f},D_{[1:K],E},A_{E}^{f^{\prime }},Q_{\left[1:N\right]}^{f^{\prime }},Z^{\prime }\right)=o\left(L\right), \end{array}$$

where the first equality follows from (7), and the second equality follows from (9). Finally, (18) holds because $W^{f^{\prime }}$ is independent from the queries and randomness, the security constraint and that $f,f^{\prime }\in GF_{q}^{K}$ are linear independent vectors. □

The following lemma shows that any answers in a set are independent from any queries conditioned on the same set of queries to the same coefficient and any size of messages and randomnesses. This is the direct inference from the independence of message, queries, and randomnesses generated by the data collector (6).

**Lemma** **2.** *Assume that $f\in GF_{q}^{N}$, $N_{1},N_{2}\in \left[1:N\right]$, and K∈[1:K]. Then, we have the following equality:*

(19) $$\begin{array}{c} I(A_{N_{1}}^{f};Q_{N_{2}}^{f}|W_{K},Z^{\prime },Q_{N_{1}}^{f})=0. \end{array}$$

**Proof.**  The proof is the same as Section VI, Lemma 1, in [29], and the key to this proof is that $A_{N_{1}}^{f}$ is determined by $W_{\left[1:K\right]}$, conditioned on $Z^{\prime }$ and $Q_{N_{1}}^{f}$. We omit the detailed proof here. □

The lemma below has a similar form to Lemma 2, and it shows that any set of answers with size of *E* do not depend on the desired statistic, conditioned on the same set of queries and the randomnesses generated by the data collector.

**Lemma** **3.** *For any E⊆[1:N],|E|=E,*

(20) $$\begin{array}{cc} H(A_{E}^{f}|Q_{E}^{f},W^{f},Z^{\prime })= & H(A_{E}^{f}|Q_{E}^{f},Z^{\prime }). \end{array}$$

**Proof.**  We only need to show that $I(A_{E}^{f};W^{f}|Q_{E}^{f},Z^{\prime })$ is less than or equal to 0 because of its non-negativity.

$$\begin{array}{cc} I(A_{E}^{f};W^{f}|Q_{E}^{f},Z^{\prime })\leq & I(A_{E}^{f},D_{[1:K],E};W^{f}|Q_{E}^{f},Z^{\prime })\\ = & I\left(D_{[1:K],E};W^{f}|Q_{E}^{f},Z^{\prime }\right)+I\left(A_{E}^{f};W^{f}|D_{[1:K],E},Q_{E}^{f},Z^{\prime }\right) \end{array}$$

(21) $$\begin{array}{cc} = & I(D_{[1:K],E};W^{f}|Q_{E}^{f},Z^{\prime })\\ = & H\left(D_{[1:K],E}|Q_{E}^{f},Z^{\prime }\right)-H\left(D_{[1:K],E}|W^{f},Q_{E}^{f},Z^{\prime }\right) \end{array}$$

(22) $$\begin{array}{c} =0, \end{array}$$

where (21) holds because the answers $A_{E}^{f}$ are determined by $\left(D_{[1:K],E},Q_{E}^{f},Z^{\prime }\right)$ in (7), and (22) holds because of (6) and (2). □

The following lemma shows that we can split the answers into two parts—one from *E* servers that cannot decode the database and the other from N−E servers:

**Lemma** **4.** *For any $f\in GF_{q}^{K}$ and E∈[1:N], |E|=E, we obtain*

(23) $$\begin{array}{cc} \; & \left(1-\frac{E}{N}\right)H\left(A_{\left[1:N\right]}^{f}|Q_{\left[1:N\right]}^{f},Z^{\prime }\right)\geq L+H\left(A_{[1:N]/E}^{f}|W^{f},D_{[1:K],E},Q_{\left[1:N\right]}^{f},Z^{\prime }\right)-o\left(L\right). \end{array}$$

**Proof.**  Based on the system model, we have

$$\begin{array}{cc} \; & H(A_{\left[1:N\right]}^{f}|Q_{\left[1:N\right]}^{f},Z^{\prime })\\ = & H\left(W^{f}|Q_{\left[1:N\right]}^{f},Z^{\prime }\right)+H\left(A_{\left[1:N\right]}^{f}|W^{f},Q_{\left[1:N\right]}^{f},Z^{\prime }\right)-H\left(W^{f}|A_{\left[1:N\right]}^{f},Q_{\left[1:N\right]}^{f},Z^{\prime }\right) \end{array}$$

(24) $$\begin{array}{cc} = & L+H\left(A_{\left[1:N\right]}^{f}|W^{f},Q_{\left[1:N\right]}^{f},Z^{\prime }\right)-o\left(L\right)\\ = & L+H\left(A_{E}^{f}|W^{f},Q_{\left[1:N\right]}^{f},Z^{\prime }\right)+H\left(A_{[1:N]/E}^{f}|W^{f},A_{E}^{f},Q_{\left[1:N\right]}^{f},Z^{\prime }\right)-o\left(L\right) \end{array}$$

(25) $$\begin{array}{cc} = & L+H\left(A_{E}^{f}|W^{f},Q_{E}^{f},Z^{\prime }\right)+H\left(A_{[1:N]/E}^{f}|W^{f},A_{E}^{f},Q_{\left[1:N\right]}^{f},Z^{\prime }\right)-o\left(L\right) \end{array}$$

(26) $$\begin{array}{cc} = & L+H\left(A_{E}^{f}|Q_{E}^{f},Z^{\prime }\right)+H\left(A_{[1:N]/E}^{f}|W^{f},A_{E}^{f},Q_{\left[1:N\right]}^{f},Z^{\prime }\right)-o\left(L\right) \end{array}$$

(27) $$\begin{array}{cc} \geq & L+H\left(A_{E}^{f}|Q_{E}^{f},Z^{\prime }\right)+H\left(A_{[1:N]/E}^{f}|W^{f},D_{[1:K],E},Q_{\left[1:N\right]}^{f},Z^{\prime }\right)-o\left(L\right) \end{array}$$

(28) $$\begin{array}{cc} \geq & L+\frac{E}{N}H\left(A_{\left[1:N\right]}^{f}|Q_{\left[1:N\right]}^{f}\right)+H\left(A_{[1:N]/E}^{f}|W^{f},D_{[1:K],E},S,Q_{\left[1:N\right]}^{f},Z^{\prime }\right)-o\left(L\right), \end{array}$$

where (24) follows from (1), (6), and (9); (25) follows from Lemma 2 when $N_{1}=E$ and $N_{2}=\left[1:N\right]$; (26) follows from (20), (6), and (7); (27) is due to (7); and (28) follows from the Han’s inequality—i.e.,

(29) ∑E⊆[1:N],|E|=EH(AEf|QEf)≥ENNEH(A[1:N]f|Q[1:N]f).

 □

Now, we can get the lower bound on the asymptotic download size when *L* and *K* goes infinity by applying (14) to (23). We have

$$\begin{array}{cc} \; & \lim_{K\to \infty ,L\to \infty }\left(1-\frac{E}{N}\right)H\left(A_{\left[1:N\right]}^{f}|Q_{\left[1:N\right]}^{f}\right) \end{array}$$

(30) $$\begin{array}{cc} \geq & \lim_{K\to \infty ,L\to \infty }\left(H\left(A_{[1:N]/E}^{f}|W^{f},D_{[1:K],E},S,Q_{\left[1:N\right]}^{f}\right)+L-o\left(L\right)\right) \end{array}$$

(31) $$\begin{array}{cc} = & \lim_{K\to \infty ,L\to \infty }H\left(A_{[1:N]/E}^{f}|W^{f},D_{[1:K],E},S,Q_{\left[1:N\right]}^{f}\right)+L\\ \geq & \lim_{K\to \infty ,L\to \infty }\frac{1}{N-E}\left(L+H(A_{[1:N]/E}^{f^{\prime }}|W^{f},W^{f^{\prime }},D_{[1:K],E},S,Q_{\left[1:N\right]}^{f^{\prime }}-o\left(L\right))\right)+L \end{array}$$

(32) $$\begin{array}{cc} \geq & \left(\sum_{k=0}^{\infty }\frac{1}{{\left(N-E\right)}^{k}}\right)L, \end{array}$$

where (30) follows from (23), (31) is because o(L) goes zero when L→∞, and (32) follows from the non-negativity of the conditional entropy. The iteration (14) can be continued because for any k∈N, $\frac{o(L)}{{\left(N-E\right)}^{k}}\to 0$ when L→∞. Thus, we can calculate the upper bound of the asymptotic capacity when E<N−1 as follows:

$$\begin{array}{cc} \lim_{K\to \infty ,L\to \infty }C\leq & \lim_{K\to \infty ,L\to \infty }\frac{L}{H(A_{\left[1:N\right]}^{f})}\\ \leq & \lim_{K\to \infty ,L\to \infty }\frac{L}{H(A_{\left[1:N\right]}^{f}|H\left(Q_{\left[1:N\right]}^{f}\right)}\\ \leq & \frac{1-\frac{E}{N}}{\sum_{k=0}^{\infty }\frac{1}{{\left(N-E\right)}^{k}}}\\ = & \frac{N-E-1}{N}. \end{array}$$

Thus, for any possible scheme ϕ satisfying the constraints of the problem, the rate cannot be more than $\frac{N-E-1}{N}$ when E<N−1 and K→∞.

## 5. Proof of Theorem 1: Achievability When E<N−1

In this section, we give a cross-subspace alignment (CSA) scheme based on the coding of interference in the computation phase to reach the asymptotic capacity [33] for any integers N>E+1 and K≥2. Throughout the scheme, we choose the length of each personal message L=N−E−1≥1, and we use the notation $\Delta_{n}=\prod_{i=1}^{L}\left(i+\alpha_{n}\right)$ for n∈[1:N].

First, we specify the encoding functions ${\left\{d_{k}\right\}}_{k\in [1:K]}$ in the upload phase. Let $W_{k}^{l}\in GF_{q}$ be the *l*-th symbol of each $W_{k}$, k∈[1:K],l∈[1:L], and $W^{l}\in GF_{q}^{1\times K}$ be the row vector of the *l*-th symbol of all *K* messages, i.e., $W^{l}=\left[W_{1}^{l},\cdots ,W_{K}^{l}\right]$. Assume that $\alpha_{n},n\in \left[1:N\right]$ are *N* distinct coefficients all belonging to the set $\left\{\alpha \in GF_{q}:\alpha +i\neq 0,i\in \left[1:L\right]\right\}$—i.e., for any $i,j\in \left[1:N\right],\alpha_{i}\neq \alpha_{j}$. Note that the $\alpha_{n}$s are globally shared variables known to the users, servers, and the data collector. In order to protect the privacy of the users against the servers, each user *k* will generate L×E random noises $Z_{le}^{k}$ uniformly from $GF_{q}$. The uploaded information to the *n*-th server by the *k*-th user is given by

(33) $$\begin{array}{c} D_{k,n}=d_{k,n}\left(W_{k},Z_{k}\right)={\left[\begin{array}{c} W_{k}^{1}+\sum_{e=1}^{E}{\left(1+\alpha_{n}\right)}^{e}Z_{1e}^{k}\\ \vdots \\ W_{k}^{L}+\sum_{e=1}^{E}{\left(L+\alpha_{n}\right)}^{e}Z_{Le}^{k} \end{array}\right]}^{T},\;\forall k\in \left[1:K\right],n\in \left[1:N\right], \end{array}$$

For convenience of notation, we write the content stored at server *n* in a vector form as

(34) $$\begin{array}{cc} D_{n}= & \left[D_{1,n}^{1},\cdots ,D_{K,n}^{1},D_{1,n}^{2},\cdots ,D_{K,n}^{2},\cdots ,D_{1,n}^{L},\cdots ,D_{K,n}^{L}\right] \end{array}$$

(35) $$\begin{array}{cc} = & {\left[\begin{array}{c} W^{1}+\sum_{e=1}^{E}{\left(1+\alpha_{n}\right)}^{e}Z_{1e}\\ \vdots \\ W^{L}+\sum_{e=1}^{E}{\left(L+\alpha_{n}\right)}^{e}Z_{Le}. \end{array}\right]}^{T}, \end{array}$$

where $D_{n}\in GF_{q}^{1\times KL}$, and $Z_{le}$ is defined as $Z_{le}=\left[Z_{le}^{1},\cdots ,Z_{le}^{K}\right]$.

In the computation phase, the query to Server *n* is determined by the coefficient f and the randomness from the data collector $Z^{\prime }$. We design the query to Server *n* based on f as

(36) $$\begin{array}{c} Q_{n}^{f}=\left[\begin{array}{c} \frac{\Delta_{n}}{1+\alpha_{n}}\left(f+\left(1+\alpha_{n}\right)Z_{1}^{\prime }\right)\\ \vdots \\ \frac{\Delta_{n}}{L+\alpha_{n}}\left(f+\left(L+\alpha_{n}\right)Z_{L}^{\prime }\right) \end{array}\right], \end{array}$$

where $Z_{1}^{\prime },\cdots ,Z_{L}^{\prime }$ are *L* random column vectors of length *K*, whose elements are uniformly distributed on $GF_{q}$, generated by the data collector.

For any server n∈[1:N], the answer to the data collector $A_{n}^{f}\in A_{n}=GF_{q}$ is calculated by

(37) $$\begin{array}{cc} \; & A_{n}^{f}=a_{n}^{f}\left(Q_{n}^{f},D_{[1:K],n}\right)=D_{n}\cdot Q_{n}^{f}\\ = & \left(W^{1}+\sum_{e=1}^{E}{\left(1+\alpha_{n}\right)}^{e}Z_{1e}\right)\cdot \left(\frac{\Delta_{n}}{1+\alpha_{n}}\left(f+\left(1+\alpha_{n}\right)Z_{1}^{\prime }\right)\right) \end{array}$$

(38) $$\begin{array}{cc} \; & +\cdots +\left(W^{L}+\sum_{e=1}^{E}{\left(L+\alpha_{n}\right)}^{e}Z_{Le}\right)\cdot \left(\frac{\Delta_{n}}{L+\alpha_{n}}\left(f+\left(L+\alpha_{n}\right)Z_{L}^{\prime }\right)\right),\forall n\in \left[1:N\right]. \end{array}$$

According to the expansion of the representation (38) in the descending power of α, we can see that $\frac{A_{n}^{f}}{\Delta_{n}}$ is the sum of $\sum_{l=1}^{L}\frac{1}{l+\alpha_{n}}W^{l}\cdot f$ and a polynomial of degree *E* in $\alpha_{n}$. We can rewrite (38) as

(39) $$\begin{array}{c} A_{n}^{f}=\Delta_{n}\left(\sum_{l=1}^{L}\frac{W^{l}f}{l+\alpha_{n}}+\sum_{e=0}^{E}I_{e}\alpha_{n}^{e}\right), \end{array}$$

where $I_{e}$ is the coefficient of $\alpha_{n}^{e}$ for any e∈[0:E]. We can clearly see from (38) and (39) that $I_{e}$ is not a function of *n*. If we write the answers to the data collector from all the *N* servers together, we can get the following formula in a matrix form:

(40) $$\left[\begin{array}{c} \frac{A_{1}^{f}}{\Delta_{1}}\\ \frac{A_{2}^{f}}{\Delta_{2}}\\ \cdots \\ \frac{A_{N}^{f}}{\Delta_{N}} \end{array}\right]=\left[\begin{array}{c} \frac{1}{1+\alpha_{1}}\;\cdots \;\frac{1}{L+\alpha_{1}}\;1\;\alpha_{1}\;\cdots \;\alpha_{1}^{E}\\ \frac{1}{1+\alpha_{2}}\;\cdots \;\frac{1}{L+\alpha_{2}}\;1\;\alpha_{2}\;\cdots \;\alpha_{2}^{E}\\ \cdots \\ \frac{1}{1+\alpha_{N}}\;\cdots \;\frac{1}{L+\alpha_{N}}\;1\;\alpha_{N}\;\cdots \;\alpha_{N}^{E} \end{array}\right]\cdot \left[\begin{array}{c} W^{1}\cdot f\\ \vdots \\ W^{L}\cdot f\\ I_{0}\\ \vdots \\ I_{E} \end{array}\right].$$

Now, we prove that this scheme satisfies the decodability constraint, i.e., (9), and the privacy constraints—i.e., the privacy constraint of the users against *E* servers (2), the privacy constraint of the users against the data collector (10), and the privacy constraint of the data collector against the non-colluding servers (11).

Recall that in our scheme, we let L=N−E−1. The decodability constraint is satisfied because the matrix in (40) is an N×N full-rank matrix when the $\alpha_{n}$s are distinct Lemma 5 in [33]. Hence, $W^{1}\cdot f,\cdots ,W^{L}\cdot f$ can be fully recovered from $\frac{A_{1}^{f}}{\Delta_{1}},\cdots ,\frac{A_{N}^{f}}{\Delta_{N}}$, and the desired data $W^{f}$ in (4) is obtained by

(41) $$\begin{array}{c} W^{f}=W^{T}\cdot f=\left[\begin{array}{c} W^{1}\cdot f\\ \cdots \\ W^{L}\cdot f \end{array}\right]. \end{array}$$

The privacy constraint of the users against *E* colluding servers is satisfied due to the sharing strategy of the users. In (33), we know that the *k*-th user shares its *l*-th symbol to the *n*-th server in a form

(42) $$\begin{array}{c} D_{k,n}^{l}=W_{k}^{l}+\sum_{e=1}^{E}{\left(1+\alpha_{n}\right)}^{e}Z_{le}^{k}, \end{array}$$

where $D_{k,n}^{l}$ denotes the storage in server *n* that $W_{k}^{l}$ shares. The security needs to guarantee that any *E* out of *N* servers do not know $W_{k}$ for any k∈[1:K]. For any set $E:=\{o_{1},\cdots ,o_{E}\}$ such that E∈[1:N] and |E|=E, we choose the *E* servers to be in E. We can write the storage of these servers with respect to what $W_{k}^{l}$ shares in a matrix form:

(43) $$\left[\begin{array}{c} D_{k,o_{1}}^{l}\\ \cdots \\ D_{k,o_{E}}^{l} \end{array}\right]=\left[\begin{array}{c} W_{k}^{l}\\ \cdots \\ W_{k}^{l} \end{array}\right]+\left[\begin{array}{c} l+\alpha_{o_{1}}\;{\left(l+\alpha_{o_{1}}\right)}^{2}\;\cdots \;{\left(l+\alpha_{o_{1}}\right)}^{E}\\ \cdots \\ l+\alpha_{o_{E}}\;{\left(l+\alpha_{o_{E}}\right)}^{2}\;\cdots \;{\left(l+\alpha_{o_{E}}\right)}^{E} \end{array}\right]\cdot \left[\begin{array}{c} Z_{l1}^{k}\\ \cdots \\ Z_{lE}^{k} \end{array}\right].$$

Notice that the Vandermonde matrix in (43), denoted by $V_{E}$, is invertible for distinct $\left\{1+\alpha_{e}:\alpha_{e}\in GF_{q},e\in E\right\}$, so the second term of (43) contains *E* symbols that are linearly independent. The privacy constraint of the users against *E* colluding servers can be guaranteed as the *E* additional random symbols protect the shared message. We have

$$\begin{array}{cc} \; & I(D_{k,E};W_{k})\\ \leq & \sum_{i=1}^{L}\sum_{j=1}^{L}I\left(D_{k,E}^{i};W_{k}^{j}|D_{k,E}^{\left[1:i-1\right]};W_{k}^{\left[1:j-1\right]}\right) \end{array}$$

(44) $$\begin{array}{cc} = & \sum_{i=1}^{L}\sum_{j=1}^{L}I\left(W_{k}^{i}1_{E}+V_{E}\cdot Z_{i,E}^{k};W^{j}|D_{[1:i-1],E};W^{\left[1:j-1\right]}\right) \end{array}$$

(45) $$\begin{array}{cc} = & \sum_{l=1}^{L}I\left(W_{k}^{l};W_{k}^{l}1_{E}+V_{E}\cdot Z_{l,E}^{k}\right) \end{array}$$

$$\begin{array}{cc} = & \sum_{l=1}^{L}I\left(W_{k}^{l};Z_{l,E}^{k}\right)\\ = & 0,\;\;\forall k\in [1:K],n\in [1:N]. \end{array}$$

where (44) comes from (43), and (45) holds because when i<j, we have

$$\begin{array}{cc} \; & I(W_{k}^{i}1_{E}+V_{E}\cdot Z_{i,E}^{k};W^{j}|D_{[1:i-1],E};W^{\left[1:j-1\right]})\\ = & H\left(W_{k}^{i}1_{E}+V_{E}\cdot Z_{i,E}^{k}|D_{[1:i-1],E};W^{\left[1:j-1\right]}\right)-H\left(W_{k}^{i}1_{E}+V_{E}\cdot Z_{i,E}^{k}|D_{[1:i-1],E};W^{\left[1:j\right]}\right)\\ = & H\left(W_{k}^{i}1_{E}+V_{E}\cdot Z_{i,E}^{k}|W^{i}\right)-H\left(W_{k}^{i}1_{E}+V_{E}\cdot Z_{i,E}^{k}|W^{i}\right)\\ = & 0,\;\forall i\in [1:L],\forall j\in [i+1,L], \end{array}$$

and when i>j, we have

$$\begin{array}{cc} \; & I(W_{k}^{i}1_{E}+V_{E}\cdot Z_{i,E}^{k};W^{j}|D_{[1:i-1],E};W^{\left[1:j-1\right]})\\ = & H\left(W_{k}^{i}1_{E}+V_{E}\cdot Z_{i,E}^{k}|D_{[1:i-1],E};W^{\left[1:j-1\right]}\right)-H\left(W_{k}^{i}1_{E}+V_{E}\cdot Z_{i,E}^{k}|D_{[1:i-1],E};W^{\left[1:j\right]}\right)\\ = & H\left(W_{k}^{i}1_{E}+V_{E}\cdot Z_{i,E}^{k}\right)-H\left(W_{k}^{i}1_{E}+V_{E}\cdot Z_{i,E}^{k}\right)\\ = & 0,\;\forall i\in [1:L],\forall j\in [1:i-1], \end{array}$$

so the remaining items are those (i,j) such that i=j=l.

To prove that the privacy constraint of the data collector against the non-colluding servers is satisfied, we notice that the query to each server is composed of the desired coefficient f and independent additional randomness $Z_{\left[1:L\right]}^{\prime }$. Consider two linear independent vectors, $f,f^{\prime }\in GF_{q}^{K}$. For any l∈[1:L], the *l*-th entries of $Q_{n}^{f}$ and $Q_{n}^{f^{\prime }}$ are $\frac{\Delta_{n}}{l+\alpha_{n}}\left(f+\left(l+\alpha_{n}\right)Z_{l}^{\prime }\right)$ and $\frac{\Delta_{n}}{l+\alpha_{n}}\left(f^{\prime }+\left(l+\alpha_{n}\right)Z_{l}^{\prime }\right)$, respectively. Notice that $Z_{l}^{\prime }$ (thus $\frac{\Delta_{n}}{l+\alpha_{n}}\cdot Z_{l}^{\prime }$) is chosen uniformly from $GF_{q}^{K}$, and that $\frac{\Delta_{n}}{l+\alpha_{n}}\cdot f$ and $\frac{\Delta_{n}}{l+\alpha_{n}}\cdot f^{\prime }$ are two deterministic vectors in $GF_{q}^{K}$. The *l*-th symbol of $Q_{n}^{f}$ or $Q_{n}^{f^{\prime }}$ has the same distribution. Therefore, any single server can not distinguish queries from the data collector with one coefficient f. Furthermore, if we assume the desired coefficient t is a random variable with some distribution known only to the data collector, and f is an implementation of t, we can prove that the mutual information between t and $\left(Q_{n}^{t},A_{n}^{t},W_{\left[1:K\right]}\right)$ is zero. We have

$$\begin{array}{cc} \; & I(Q_{n}^{t},A_{n}^{t},W_{\left[1:K\right]};t) \end{array}$$

(46) $$\begin{array}{cc} \leq & I(Q_{n}^{t},W_{\left[1:K\right]},{\left\{Z_{le}\right\}}_{l\in [1:L],e\in [1:E]};t) \end{array}$$

(47) $$\begin{array}{cc} = & I(Q_{n}^{t};t|W_{\left[1:K\right]},{\left\{Z_{le}\right\}}_{l\in [1:L],e\in [1:E]}) \end{array}$$

$$\begin{array}{cc} = & H\left(Q_{n}^{t}|W_{\left[1:K\right]},{\left\{Z_{le}\right\}}_{l\in [1:L],e\in [1:E]}\right)-H\left(Q_{n}^{t}|t,W_{\left[1:K\right]},{\left\{Z_{le}\right\}}_{l\in [1:L],e\in [1:E]}\right) \end{array}$$

(48) $$\begin{array}{cc} = & H\left(Q_{n}^{t}\right)-H\left(\left.\left[\begin{array}{c} t+\left(1+\alpha_{n}\right)\left(Z_{1}^{\prime }\right)\\ \cdots \\ t+\left(L+\alpha_{n}\right)\left(Z_{L}^{\prime }\right) \end{array}\right]\right|t,W_{\left[1:K\right]},{\left\{Z_{le}\right\}}_{l\in [1:L],e\in [1:E]}\right) \end{array}$$

$$\begin{array}{cc} = & H\left(Q_{n}^{t}\right)-H\left(Z_{1}^{\prime },\cdots ,Z_{L}^{\prime }\right)\\ = & L-L\\ = & 0, \end{array}$$

where (46) is from (38), (47) is from (10), and (48) is from (6).

Finally, to prove that the privacy constraint of the users against the data collector is satisfied, we construct a basis of $GF_{q}^{K}$ containing the desired f. Assume that the vectors in the basis is denoted by $\left\{f_{1},f_{2},\cdots ,f_{K}\right\}$ where $f_{1}=f$. We then have

$$\begin{array}{cc} \; & I\left(W_{\left[1:K\right]};A_{\left[1:N\right]}^{f},Q_{\left[1:N\right]}^{f},Z^{\prime }|W^{f}\right)\\ = & \sum_{l=1}^{L}I\left(W^{l};A_{\left[1:N\right]}^{f},Q_{\left[1:N\right]}^{f},Z^{\prime }|W^{\left[1:l-1\right]},W^{f}\right) \end{array}$$

(49) $$\begin{array}{cc} \leq & \sum_{l=1}^{L}I\left(W^{l};A_{\left[1:N\right]}^{f},Q_{\left[1:N\right]}^{f},Z^{\prime }|W^{\left[1:L]/\{l\right\}},Z,W^{f}\right)\\ = & \sum_{l=1}^{L}I\left(\left(W^{l}+\sum_{e=1}^{E}{\left(l+\alpha_{n}\right)}^{e}Z_{le}\right)\cdot \right. \end{array}$$

(50) $$\begin{array}{cc} \; & \;\;\;\;\left.{\left(\frac{\Delta_{n}}{1+\alpha_{n}}\left(f+\left(l+\alpha_{n}\right){\left(Z_{l}^{\prime }\right)}^{T}\right)\right)}_{n\in [1:N]};W^{l}|W^{\left[1:L]/\{l\right\}},Z,W^{f}\right) \end{array}$$

(51) $$\begin{array}{cc} = & \sum_{l=1}^{L}I\left({\left(W^{l}{\left(Z_{l}^{\prime }\right)}^{T}+\sum_{e=1}^{E}{\left(l+\alpha_{n}\right)}^{e}Z_{le}{\left(Z_{l}^{\prime }\right)}^{T}\right)}_{n\in [1:N]};W^{l}|W^{\left[1:L]/\{l\right\}},W^{f}\right) \end{array}$$

(52) $$\begin{array}{cc} \leq & \sum_{l=1}^{L}I\left({\left(W^{l}{\left(Z_{l}^{\prime }\right)}^{T},Z_{le}{\left(Z_{l}^{\prime }\right)}^{T}\right)}_{e\in [1:E]};W^{l}|W^{\left[1:L]/\{l\right\}},W^{f}\right) \end{array}$$

(53) $$\begin{array}{cc} = & 0, \end{array}$$

where (49) holds because $\left(W^{\left[1:L]/\{l\right\}},Z\right)$ is independent with $W^{l}$; (50) holds because except for the term containing $W^{l}$, all terms in (38) are given, so deducting them would not change the mutual information; (51) is because $\Delta_{n}$ is a constant; (52) is because for any n∈[1:N] and e∈[1:E], ${\left(l+\alpha_{n}\right)}^{e}$ is a constant, and for n∈[1:N], $W^{l}{\left(Z_{l}^{\prime }\right)}^{T}+\sum_{e=1}^{E}{\left(l+\alpha_{n}\right)}^{e}Z_{le}{\left(Z_{l}^{\prime }\right)}^{T}$ can be derived by $W^{l}{\left(Z_{l}^{\prime }\right)}^{T}$ and ${\left(Z_{le}{\left(Z_{l}^{\prime }\right)}^{T}\right)}_{e\in [1:E]}$; and (53) holds because for any l∈[1:L], ${\left\{Z_{le}\right\}}_{e\in [1:E]}$ and $Z_{l}^{\prime }$ are generated independently of $W^{\left[1:L\right]}$.

Thus, we prove that the scheme satisfies all the constraints. As the answer from any server contains one symbol (the inner product) from $GF_{q}$, the rate of our proposed scheme is

(54) $$\begin{array}{c} R=\frac{L}{\sum_{n=1}^{N}H\left(A_{n}^{f}\right)}\frac{N-E-1}{N}. \end{array}$$

It can be seen that the scheme we construct by (33), (36), and (38) is achievable with the rate (54) invariant with *K* by rearranging the data $W_{\left[1:K\right]}$ to $W^{\left[1:L\right]}$. We notice that the achievable rate meets the asymptotic upper bound for any $K\in N_{+}$, so the scheme is then proved to be asymptotically optimal, by letting K→∞.

## 6. Converse Result When E=N−1

In this section, we prove that the asymptotic capacity is zero when N=E+1. First, the inequality (23) also holds when N=E+1 because the inequality in (15) becomes an equality. Hence, we have

(55) $$\begin{array}{cc} H(A_{[1:N]/E}^{f}|W^{f},D_{[1:K],E},S,Q_{\left[1:N\right]}^{f},Z^{\prime })\geq & L+H\left(A_{[1:N]/E}^{f^{\prime }}|W^{f},W^{f^{\prime }},D_{[1:K],E},Q_{\left[1:N\right]}^{f^{\prime }},Z^{\prime }\right)-o\left(L\right). \end{array}$$

Thus, for any linear independent vectors $f_{1},f_{2},\cdots ,f_{K}\in GF_{q}^{K}$, we have

(56) $$\begin{array}{cc} \left(1-\frac{E}{N}\right)H\left(A_{\left[1:N\right]}^{f}|Q_{\left[1:N\right]}^{f}\right)\geq & H\left(A_{[1:N]/E}^{f_{1}}|W^{f_{1}},D_{[1:K],E},Q_{\left[1:N\right]}^{f_{1}},Z^{\prime }\right)+L-o\left(L\right) \end{array}$$

(57) $$\begin{array}{cc} \geq & 2L+H\left(A_{[1:N]/E}^{f_{2}}|W^{f_{1}},D_{[1:K],E},Q_{\left[1:N\right]}^{f_{2}},Z^{\prime }\right)-o\left(L\right) \end{array}$$

(58) $$\begin{array}{cc} \geq & \sum_{k=0}^{K}L+H\left(A_{[1:N]/E}^{f_{K}}|W^{\left[1:L\right]},D_{[1:K],E},Q_{\left[1:N\right]}^{f_{K}},Z^{\prime }\right) \end{array}$$

(59) $$\begin{array}{cc} = & \sum_{k=0}^{K}L+H\left(A_{[1:N]/E}^{f_{K}}|W_{\left[1:K\right]},D_{[1:K],E},Q_{\left[1:N\right]}^{f_{K}},Z^{\prime }\right)\\ = & KL, \end{array}$$

where (56), (57), and (58) are from (55); and (59) holds because any *K* linear independent vectors constitute a basis in $GF_{q}^{K}$, so $W_{\left[1:K\right]}$ can be decoded. We can see that the download will go to infinity when K→∞, and thus the asymptotic capacity will be

$$\begin{array}{cc} \lim_{K\to \infty ,L\to \infty }C\leq & \lim_{K\to \infty ,L\to \infty }\frac{L}{H(A_{\left[1:N\right]}^{f})}\\ \leq & \lim_{K\to \infty ,L\to \infty }\frac{L}{H(A_{\left[1:N\right]}^{f}|H\left(Q_{\left[1:N\right]}^{f}\right)}\\ \leq & \lim_{K\to \infty ,L\to \infty }\frac{1-\frac{E}{N}}{KL}\\ = & 0. \end{array}$$

The upper bound of *C* indicates that it is unfeasible to construct a scheme that has a positive communication rate when K→∞. However, differently from the N=E scenario, our proof of the zero asymptotic capacity does not mean that there does not exist any scheme that satisfies all the constraints of our problem. In other words, there may be schemes that can satisfy all the constraints of the problem, albeit with an asymptotic capacity of zero. The detailed construction of a feasible scheme for E=N−1 and finite *K* is still an open problem.

## 7. Conclusions

Thanks to the research on data privacy and modeling of infectious diseases, the privacy-preserving epidemiological-data-collection problem was proposed, which aims to maximize the collection rate while protecting the privacy of all users’ data and the data collector’s preferences. We have found the asymptotic capacity of this problem, and the result shows that when there is more than one remaining server that not colluding with the other servers to decode the users’ data, the asymptotic capacity exists. The objective of this work was to find the best performance for the privacy-preserving epidemiological-data-collection problem, and we partly achieved this goal by giving the construction and proof of the optimal scheme when *K* is infinitely large. The achievability for N≥E+2 was given by the cross-subspace alignment method, and the infeasibility of N=1 or N=E was also proved. Our findings not only extend the research on secure multi-party computing systems in information theory, but also provide information-theoretic frameworks, implementations, and capacity bounds for the study of privacy epidemiological modeling. Although we characterized the asymptotic capacity for this problem, the exact capacity is still unknown. Some future directions include finding the exact capacity for finite *K*, the construction of a scheme when N=E+1, and the performance of asymptotic capacity under irregular colluding patterns. In general, our result of asymptotic capacity for this problem will provide useful insights for further studies in data both privacy and epidemiological modeling.

## Data Availability

Data sharing is not applicable to this article as no new data were created or analyzed in this study.

## Abbreviations

The following abbreviations are used in this manuscript:

COVID-19	Corona Virus Disease 2019
GSM	Global System for Mobile Communications
P-AEEF	Privacy-Aware Energy-Efficient Framework
LSTM	Long Short-Term Memory
CNN	Convolutional Neural Network
CSA	Cross Subspace Alignment
